# Operationalising the “One Health” approach in India: facilitators of and barriers to effective cross-sector convergence for zoonoses prevention and control

**DOI:** 10.1186/s12889-021-11545-7

**Published:** 2021-08-06

**Authors:** F. A. Asaaga, J. C. Young, M. A. Oommen, R. Chandarana, J. August, J. Joshi, M. M. Chanda, A. T. Vanak, P. N. Srinivas, S. L. Hoti, T. Seshadri, B. V. Purse

**Affiliations:** 1 UK Centre for Ecology & Hydrology, Wallingford, OX10 8BB UK; 2 UK Centre for Ecology & Hydrology, Edinburgh, EH26 0QB UK; 3 Agroécologie, AgroSup Dijon, INRAE, Univ. Bourgogne, Univ. Bourgogne Franche-Comté, F-21000 Dijon, France; 4 Ashoka Trust for Research in Ecology and the Environment, Bengaluru, 560 054 India; 5 Oxford Brookes University, Headington Campus, Oxford, OX3 0BP UK; 6 Centre for Disease Dynamics, Economics & Policy, B-25, Lajpat Nagar-2, New Delhi, India; 7 ICAR-National Institute of Veterinary Epidemiology and Disease Informatics, Ramagondanahalli, Yelahanka New Town, Bengaluru, Karnataka 560064 India; 8 School of Life Sciences, University of KwaZulu-Natal, Pietermaritzburg, 3209 South Africa; 9 DBT-Wellcome Trust India Alliance, Hyderabad, 500034 India; 10 Institute of Public Health, Banashankari 2nd Stage, Bangalore, 560 070 India; 11 ICMR-National Institute for Traditional Medicine, Belgavi, Karnataka 590010 India

**Keywords:** One health, Cross-sectoral convergence, Emerging infectious disease, Zoonoses, Health system, India

## Abstract

**Background:**

There is a strong policy impetus for the One Health cross-sectoral approach to address the complex challenge of zoonotic diseases, particularly in low/lower middle income countries (LMICs). Yet the implementation of this approach in LMIC contexts such as India has proven challenging, due partly to the relatively limited practical guidance and understanding on how to foster and sustain cross-sector collaborations. This study addresses this gap by exploring the facilitators of and barriers to successful convergence between the human, animal and environmental health sectors in India.

**Methods:**

A mixed methods study was conducted using a detailed content review of national policy documents and in-depth semi-structured interview data on zoonotic disease management in India. In total, 29 policy documents were reviewed and 15 key informant interviews were undertaken with national and state level policymakers, disease managers and experts operating within the human-animal-environment interface of zoonotic disease control.

**Results:**

Our findings suggest that there is limited policy visibility of zoonotic diseases, although global zoonoses, especially those identified to be of pandemic potential by international organisations (e.g. CDC, WHO and OIE) rather than local, high burden endemic diseases, have high recognition in the existing policy agenda setting. Despite the widespread acknowledgement of the importance of cross-sectoral collaboration, a myriad of factors operated to either constrain or facilitate the success of cross-sectoral convergence at different stages (i.e. information-sharing, undertaking common activities and merging resources and infrastructure) of cross-sectoral action. Importantly, participants identified the lack of supportive policies, conflicting departmental priorities and limited institutional capacities as major barriers that hamper effective cross-sectoral collaboration on zoonotic disease control. Building on existing informal inter-personal relationships and collaboration platforms were suggested by participants as the way forward.

**Conclusion:**

Our findings point to the importance of strengthening existing national policy frameworks as a first step for leveraging cross-sectoral capacity for improved disease surveillance and interventions. This requires the contextual adaptation of the One Health approach in a manner that is sensitive to the underlying socio-political, institutional and cultural context that determines and shapes outcomes of cross-sector collaborative arrangements.

**Supplementary Information:**

The online version contains supplementary material available at 10.1186/s12889-021-11545-7.

## Background

In recent theoretical and policy debates on public health, the One Health^1^ (OH) movement has gained momentum and has increasingly been put forward not only as a means of surmounting the threat of zoonotic diseases but also as a viable strategy for achieving the UN Sustainable Development Goals (SDGs) [2, 3]. According to proponents of the OH movement, cross-sectoral convergence^2^ provides a window of opportunity to transition from traditional “silo-based” functioning towards an integrated approach to zoonotic diseases at the human, animal and environment interface [7]. This argument is particularly compelling given that 75% of all infectious diseases have an animal origin [8, 9], which underscores the importance of fostering this form of cross-sectoral engagement. The outbreaks of devastating epidemics such as highly pathogenic avian influenza (HPAI) (2003), Ebola (2014) and more recently the novel coronavirus (2019) have given additional impetus to the need for cross-sector engagement as exemplified by the widespread endorsement of the OH movement by leading international agencies (the Food and Agriculture Organisation, World Organisation for Animal Health, World Health Organisation) and successive national governments globally [3, 7, 10]. At the same time, the OH approach is also witnessed as crucial to effectively tackle endemic infections (such as brucellosis, leptospirosis and plague) with huge, yet under-reported disease burdens, particularly in poor marginalised populations [11–13].

While applauded as a game-changer [3, 6], the implementation of the OH approach has at best been challenging particularly in LMICs, including India [14, 15]. The reasons for such limited achievement are wide ranging [6, 14]. Several scholars have for instance, highlighted the necessity to engage wide range of relevant professionals to contribute valuable skillset and perspectives, competing health and development priorities, and limited personnel and funding capacities as some of the fundamental challenges to the effective implementation of OH initiatives [16, 17]. The international research community is thus saddled with the challenge of optimising OH implementation initiatives by producing practical/ grounded evidence that incorporates theoretical OH issues and an understanding of the contextual influences [6, 11, 18, 19]. Within this context, the FAO-OIE-WHO *Tripartite Commitment* document identified, inter alia, the need to better understand the operational and local contextual factors required to support effective OH operationalisation [20]. Whereas there is considerable theoretical evidence about the enablers and barriers to effective operationalisation of OH initiatives, there is relative dearth of corresponding empirical evidence particularly in LMIC settings [6, 21–23]. This has meant that the potential of cross-sectoral collaborations for the purposes of zoonotic disease prevention and control remain untapped in many LMICs [18, 22]. Consequently, there have been calls for more empirical studies on collaboration dynamics which may help explain the limited uptake and/or success of the OH policies and programs in developing contexts [6, 16, 24] and India specifically [25–28]. Yet, empirical studies on zoonoses control in India (with the notable exception of [8, 13, 25, 29, 30]) have tended to focus on biological and ecological determinants of disease transmission and developing technical interventions, overlooking the socio-economic, political and cultural aspects that impinge the disease system. A context-specific understanding of the dynamics of cross-sectoral collaboration within the purview of the existing governance arrangements is thus important in appropriately designing and implementing OH interventions towards more effective integrated management of endemic and emerging zoonotic diseases.

The study aims to: (1) inform the effective operationalisation of contextually appropriate OH, by improving practical understanding of the policy and local influences on OH implementation, and (2) identify barriers and facilitators linked to the prevention and control of zoonoses. India provides an excellent case to examine the dynamics of OH operationalisation for two main reasons. First, India ranks high globally in terms of the burden and diversity of endemic and emerging zoonotic diseases [31, 32]. Impacts are especially worse for poor communities, impeding poverty alleviation, food production and over-all well-being [8, 30, 32]. Secondly, there are ongoing efforts at the national and state levels towards advancing cross-sectoral action for zoonotic disease control [13, 30]. Specifically, the paper explores: (1) how policies for zoonotic diseases are organised across sectors and scales; and (2) the factors that facilitate or constrain effective cross-sectoral action for zoonotic disease control. By so doing, this paper provides insights into the dynamics of how and why cross-sectoral convergence does or does not occur at different levels, which is important in identifying feasible pathways to advance successful collaborations in LMIC settings. This paper thus challenges the assumption implicit in OH and zoonoses policy debates that, policy frameworks that facilitate cross-sectoral collaboration will lead (automatically) to successful cross-sector partnerships [13, 26, 33].

## Methods

This study adopted a mixed methods approach, using systematic document review and in-depth interviews with purposively selected key informants to understand the opportunities and barriers to operationalising OH approach to control zoonotic disease of regional or national importance in India. Against this backdrop, the systematic review was conducted to provide information about the policy context, plans and implementation of cross-sectoral collaboration for zoonoses management, and reported adhering to the PRISMA guidelines.

### Systematic review – search strategy

We developed a search protocol as part of a larger Indo-UK interdisciplinary One Health project (IndiaZooSystems), which generated a list of possible relevant keywords related to zoonoses and OH operationalisation in the context of India. For the systematic search, the online bibliographic databases of PubMed and Web of Science were used, due to their wide scope of scientific publications and multidisciplinary contents. The key terms applied in the search were “zoonoses”, “policy” and “one health”. The final search syntax used was as follows: (“zoonoses” OR “zoonotic disease” OR “emerging infectious disease”) AND (“policy” OR “management” OR “one health”) AND (“India”). We also searched relevant government ministry, NGOs and international organisation, reference listings and the internet as supplementary strategy to retrieve other grey documents. Three of the authors (FAA, JA and RC) conducted all the searches between November 2018 and July 2019.

#### Eligibility criteria and selection of studies

**Fig. 1:**
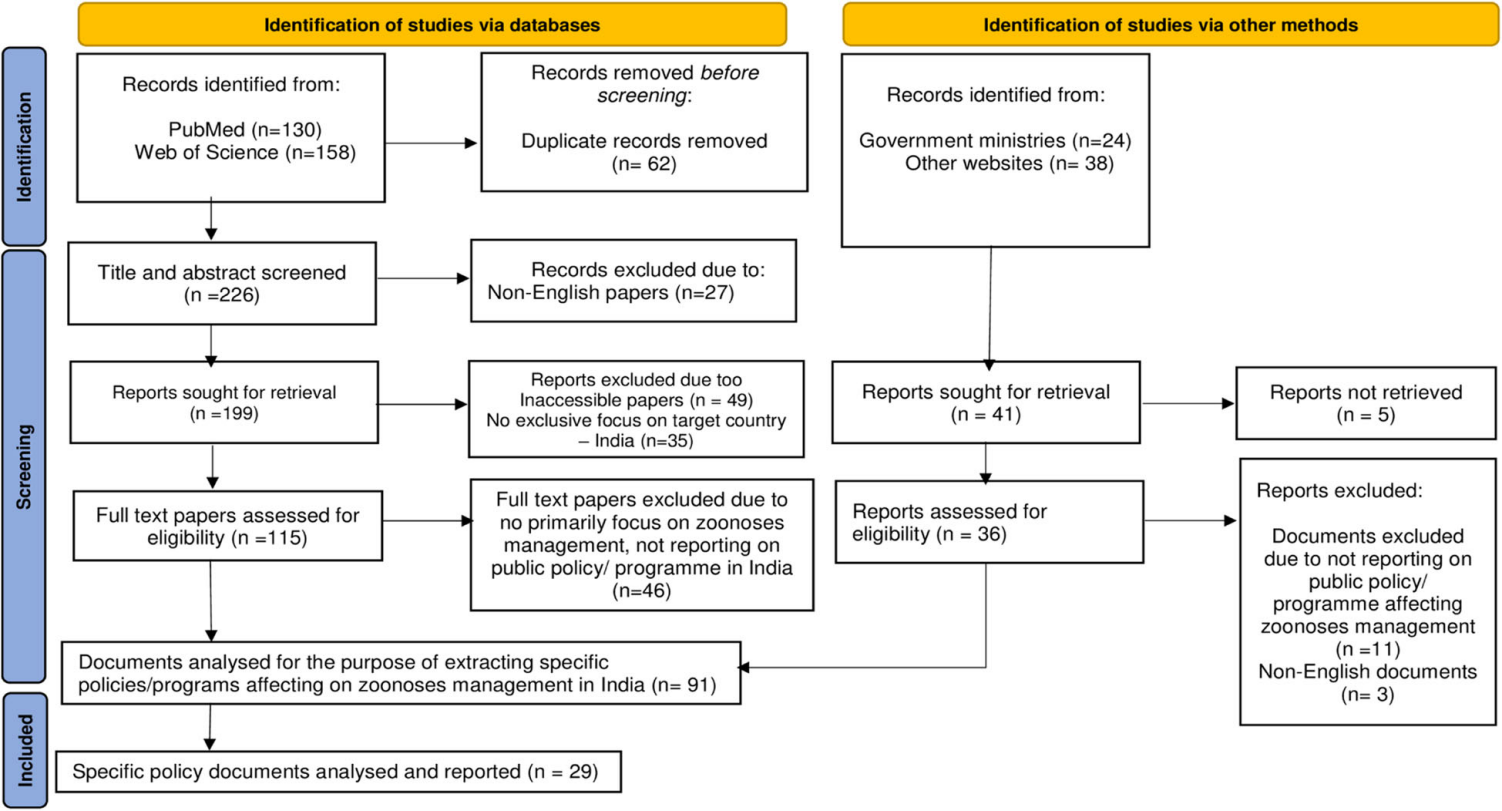
Flowchart of the selection process of relevant documents. Adapted from the Preferred Reporting Items for Systematic Review and Meta-Analysis (PRISMA) protocol by Page et al. [34]

Following the systematic search, different inclusion and exclusion criteria was used to select the papers for the review (see Fig. 1). The following inclusion criteria was applied: (1) primary focus on the subject matter – zoonotic diseases, social and environmental risk factors and disease management in Public Health, Animal Health, Forestry and Agriculture, (2) reporting a public policy or programme within the human-animal-society context in any of the sectors of interest, and (3) geographical focus on India. Papers were excluded when they did not report on policy or programme affecting zoonoses management or when they did not relate the focal country (India). The search was not restricted to a specific study design, year of publication or any other factor, except for language (English) and non-target country. The language restriction derived from the assumption that English is the official language for reporting health research in India.

Having removed duplicates that were identified from the bibliographic searches, the papers were double-screened by three reviewers (FAA, RC and JA) in two phases: first, the titles and abstract followed by the review of full text for each papers to verify that they met the afore-mentioned inclusion criteria. After undertaking the systematic search, we identified and added additional grey documents of relevance to the subject matter of this review to our dataset. In total, 151 documents were imported into an F1000 database for this review.

#### Data extraction and analysis

After the selection of studies, two reviewers (FAA and RC) systematically screened papers via a content analysis approach to specifically identify all the relevant policies, strategic plans, legislation, program guidelines and protocols reported as influencing zoonoses management in India. Based on this approach, we identified 29 specific policies and programmes on zoonoses management in India. The review team subsequently sourced 29 related policy documents (produced between 1988 and 2018 by the Indian government, NGOs, and international organisations (e.g. CDC, WHO and OIE)) that were thematically analysed using a template created in Bristol Online Surveys (BOS). We assessed information such as the primary focus of the policy/ program, level of emphasis on cross-sectoral collaboration, sectoral representation and roles, relevance to zoonoses, and coherence with the OH approach. Further semi-quantitative analysis was conducted to measure the frequency of sectoral representation, emphasis on cross-sectoral collaboration and coherence with OH approach by the sourced policy documents.

### Key informant interviews

We supplemented the review of documents and published literature on zoonoses management with a series of in-depth key informant interviews (*n* = 15) conducted (between December 2018 and August 2019) with key actors and practitioners (directly or indirectly) influencing zoonoses management in India. A total of thirty-four (34) key actors and practitioners were invited via email^3^ (with an outline of the study objectives) to participate in the research, fifteen (15) of whom agreed based on availability. The participants included officials from the union and state departments and ministries of Health & Family Welfare (MoH&FW), Agriculture & Farmers Welfare (MoA&FW), and actors from policy and practice bodies such as the Roadmap to Combat Zoonoses in India (RCZI), academia, international research institutes and non-governmental organisations (Table 1). Purposive sampling was used to select participants based on their prior experience, expertise and active involvement in zoonotic disease control and OH policy dialogue [35].

**Table 1:** Interview informants’ distribution by domain of influence and expertise

Interviewee Domain	Type of Power/ Influence	Influence on policy formulation	Number of Informants	Experience Level
Policy - Central government (Human health sector)	Decision-makers and influence policy formulation	High	3	15–26 years
Policy - State or District government (Animal health sector)	Decision-makers and influence policy formulation/ implementation	High	2	15–25 years
Policy - State or District government (Human health sector)	Decision-makers and influence policy formulation/ implementation	High	4	10–15 years
Academic or Research	Knowledge generation and influences policy formulation	Low- moderate	4	20–30 years
International representative(s)	Decision-maker and influences policy formulation	Moderate	1	13 years
Non-governmental representatives (Environment sector)	Practitioner and influences policy implementation	Low – moderate	1	12 years
**Total**			**15**	

A semi-structured interview guide was developed and included questions relating to three main areas: existing policy context, management strategies and cross-sectoral collaborations in the context of disease control (see Interview guide in Additional file 1). The same interview guide was used across all the interviews but adapted to suit the specific context of individual informants’ expertise and experience where necessary. We solicited participants’ views on the roles, existing prioritisation of zoonoses, sectoral actors involved in zoonoses management, barriers and opportunities for institutionalising OH approach in India. All the interviews were on one-to-one basis conducted in English via telephone or skype, according to interviewees’ preference. On average, the interviews lasted between 45 min and 1 h and were audio-tapped with the prior consent of participants. All interviews were transcribed and manually coded according to the emerging themes and topics, from which key narratives and storylines were developed following after Braun and Clarke’s guide to thematic analysis [36]. We read the interview transcripts repeatedly to ensure familiarisation and immersion in the data. This was followed by a line-by-line coding of each transcript by two investigators (FAA and RC) based on informants’ meanings and content. The generated codes were organised into themes, which were revised iteratively taking due cognizance of internal heterogeneity and external heterogeneity [37]. Through this iterative process, relevant themes (comprising 4 main themes and 22 sub-themes) were defined and finalised (see Table 2 and Supplementary Figure 3). The interview data were triangulated with the document review data based on which inferences and conclusions were drawn [38, 39]. Drawing on the composite interview and document data, a further thematic content analysis was conducted with a view to mapping the key stakeholders, their roles and sectoral domains, interrelationships and scale of involvement in zoonoses management in India. The ensuing paragraphs presents the observations and experience of key actors at the forefront of disease control and OH policy dialogue in India.

**Table 2:** Summary of key themes and sub-themes from the analysis

Theme	Sub-themes and frequency cited
Zoonotic disease governance	Complex organisation of the zoonotic disease governance system (15 out of 15 interviewees)
	Health as a state subject (decentralised decision-making) (14 out of 15 interviewees)
	Central government influence on state health policy agenda setting (10 out of 15 interviewees)
Political prioritisation of zoonoses	Low prioritisation of zoonoses in health policy (12 out of 15 interviewees)
	No systematic framework for disease prioritisation (10 out 15 interviewees)
	Different zoonoses have different level of recognition in existing policy agenda (13 out of 15 interviewees)
	Unsupportive policies (9 out of 15 interviewees)
Barriers to cross-sectoral action for zoonotic disease control	Disciplinary/ sectoral silos/ turf wars (12 out of 15 stakeholders)
	Disparate human and animal disease reporting/ surveillance systems (10 out of 15 interviewees)
	Communication and information asymmetries (15 out of 15 interviewees)
	Differences in disciplinary training (9 out of 15 interviewees)
	Knowledge deficits (11 out of 15 interviewees)
	Perceived mistrust, ‘egos’ and different mind-sets among actors (12 out of 15 interviewees)
	Inadequate infrastructure and funding allocation (11 out of 15 interviewees)
	Institutional bureaucracy and coordination challenges (13 out of 15 interviewees)
	Competing department priorities (11 out of 15 interviewees)
	Entrenched hierarchical system (12 out of 15 interviewees)
	Differences in regional capacities and working practices (10 out of 15 interviewees)
Facilitators of cross-sectoral action for zoonotic disease control	Formal governance and leadership structures (15 out of 15 interviewees)
	Clear delineation of sectoral roles (10 out of 15 interviewees)
	Improving communication and working relationships (15 out of 15 interviewees)
	Resourcing considerations (12 out of 15 interviewees)

## Results

### Institutional landscape for zoonoses control: identifying the ‘gatekeepers’ for OH

**Fig. 2:**
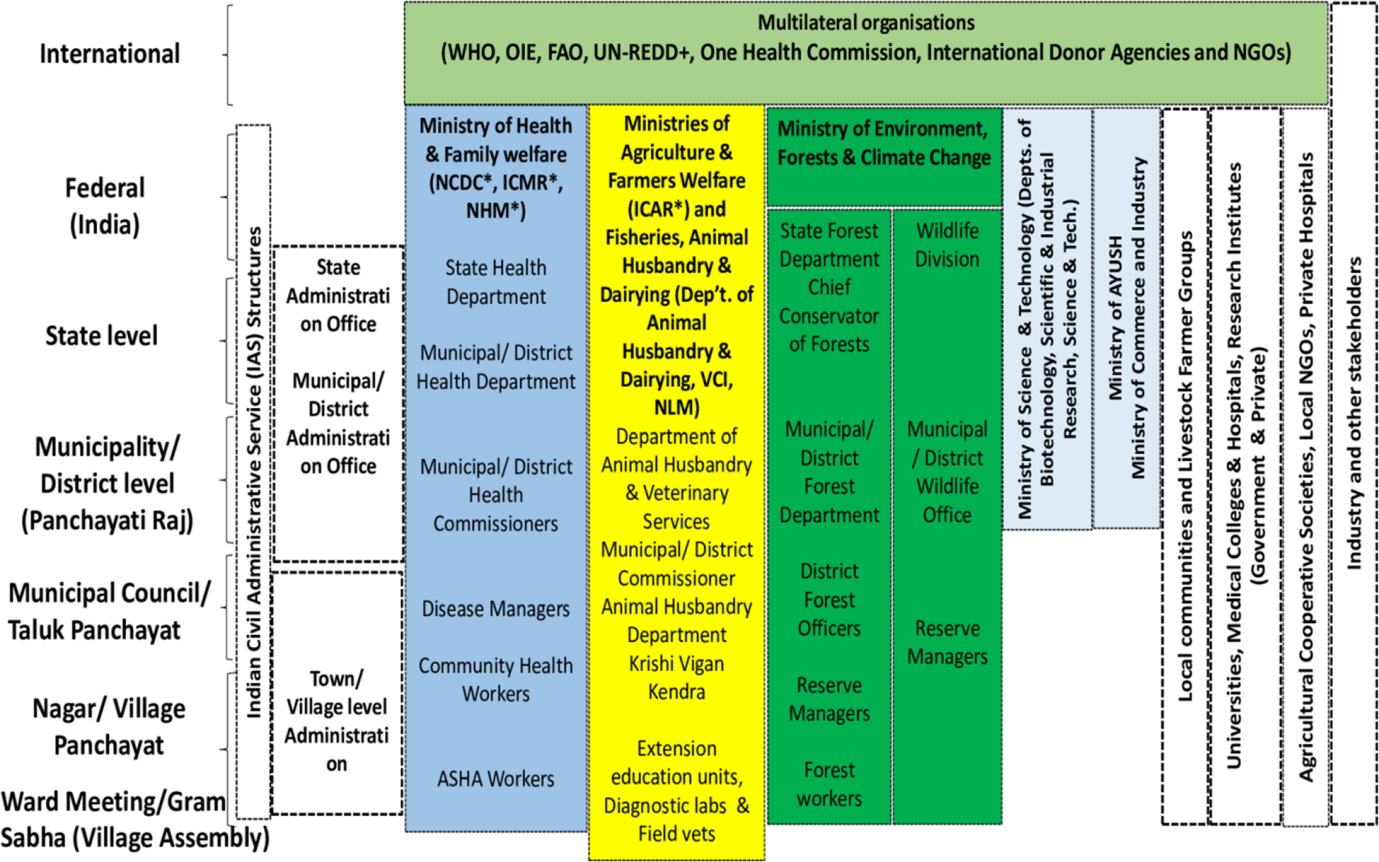
A simplified illustration of the sectors and the politico-administrative actors of the health system in relation to zoonotic disease control in India including actors from each sector that are impinging on the system or are impacted by disease in each sector. ASHA = Accredited Social Health Activist; FAO = Food and Agriculture Organisation; NCDC = National Centre for Disease Control; ICMR = Indian Council for Medical Research; NHM = National Health Mission; ICAR = Indian Council for Agricultural Research; NLM = National Livestock Mission; NGO = Non-government Organisation; OIE = Office International des Epizooties (World Organisation for Animal Health); UN-REDD = United Nations Reducing Emissions from Deforestation and Degradation Programme; VCI = Veterinary Council of India, WHO = World Health Organisation

As described in the introduction, zoonoses constitute a huge public policy concern in India given its status as a one of the global ‘hotspots’ and the large human and livestock population [8, 31]. This is exemplified by the high rate of re-emergence and high disease burdens linked to rapid socio-ecological and environmental changes [40]. Although this development has produced a significant history of response to important zoonotic disease outbreaks [30], it has also triggered a need to deal with novel epidemics as they arise. To contextualise the empirical evidence from this study, it is thus important to characterise the Indian health system, particularly the governance structure and zoonotic disease prioritisation. Health is considered a two-pronged responsibility shared between the central (federal) and state governments.^4^ While the Government of India is responsible for health policies, regulatory functions, and control of diseases and outbreaks (through the Ministry of Health and Family Welfare (MoH&FW), the state governments are tasked with healthcare and training of personnel [25]. Figure 2 developed from the key informant interviews and policy document review, illustrates the multi-scale institutional landscape around zoonoses in India (see also Supplementary Figures 1 and 2 on the detailed hierarchical organisation of the Indian human and animal health sectors).

This figure suggests well-defined, complementary roles played by different actors within the health system, with the NCDC, ICMR and ICAR (marked in asterisk - responsible for research and innovation) having the overarching responsibility for zoonoses control and prevention within this hierarchical and sectorally defined structure. In practice, however, the picture is complex and contested, with zoonotic disease response coalesced in a politicised and hierarchical environment which shapes actor roles and key collaborative outcomes. This complexity is epitomized at two levels. The first layer of the complexity relates to the somewhat silo-ed functioning of the respective agencies under three disparate sector ministries. The NCDC^5^ and ICMR are focal human health agencies under the MoH&FW promoting human well-being. By contrast, the Animal Science division of ICAR and the Department of Animal Husbandry and Dairying (AH&D) (veterinary sector) operate under the Ministries of Agriculture and Farmers Welfare (MoA&FW) and Fisheries, Animal Husbandry & Dairying, focussing on animal health to boost food production and safety. Likewise, the wildlife sector (one of the triad of OH, 41) falls under the Ministry of Environment, Forests and Climate Change (MoEF&C) responsible for environment health and conservation-related concerns. This fragmentation and disparate sectoral affiliations makes cross-sectoral convergence difficult to achieve given the differing goals and power dynamics between ministries and departments [30]. Critical views by two of our interviewees are illustrative:

*“There are always points of conflicting policy as there is no comprehensive guidelines on synergising coordination between sector agencies and departments. The decision-making is almost always political as it is technical … ”* (Interview 7, Public Health).

*“Cross-sectoral collaboration is hampered by the very nature of the different disciplinary government agencies. So they are designed to be silos. They don’t work well with reaching out to others. This is to do with the nature of the bureaucracy, where each department thinks that all expertise lies within it. That’s one challenge … ”* (Interview 3, Environment)*.*

It therefore comes as no surprise that the wildlife sector has had limited involvement in health policy deliberations relative to the human health and veterinary sectors [26, 30]. As an example, the National Standing Committee on Zoonoses (NSCZ), a health-sponsored committee convened by the NCDC, is dominated by representatives from the human health and animal health sectors with little or no representation from the wildlife sector. The somewhat skewed setup significantly constrains the committee’s effectiveness in terms of advancing integrative cross-sectoral response given that there is a strong tendency to defer the ‘politics’ of the most senior official(s) sitting on the committee, in this case a health person (Interview 2, Public Health). This constitutes a major area of engagement in advancing cross-sectoral action under OH given that nearly three fourths of emerging zoonoses such as anthrax, avian influenza and Kyasanur Forest Disease (KFD) originate from wildlife sources [26].

The second strand of the complexity of the health system relates to the considerable variation in health (animal and human) administration and capacities at the state level, which significantly affects health policy decision-making and outcomes. Although health is a state subject, the central government by virtue of its federal level power and access to funds is often able to shape and dictate the agenda to different states (Interview 1). This has both positive and negative ramifications for disease control. On the one hand, the central government’s influence yields a positive ‘win-win’ outcome as it affords the opportunity to obtain state buy-in for common disease surveillance programmes like the Integrated Disease Surveillance Programme (IDSP), and augment state-level efforts (through funding support and technical guidelines) to control disease outbreaks. Conversely, the hierarchical setup of the health system has meant that national-level agencies (often regarded as an appendage of central government authority) have high dispositional powers, the exercise of which could, in some instances, dilute support for local health (human and animal) imperatives (see Institutional bureaucracy, entrenched hierarchy and coordination challenges section, Supplementary Figures 1 and 2), and usurp the authority of state and district-level officials. A national-level health official and (state-level) non-government actor respectively had this to say:

“*Health is not a priority for some states and health department (at the state level) is starved of funds so if the centre comes with funds and some guidelines which is evidence-informed, states are more than happy to follow them, even if it means shifting focus from other local level priorities … ”*(Interview 1, Public Health).

*“Because of the hierarchy, the (health system’s) self-feedback mechanisms are very weak. So very rarely do people sitting in Delhi and in state capitals listen to what is happening on the ground level. Very rarely do ground level people have the courage or bravery to give feedback to their seniors … ”* (Interview 3, Environment)*.*

### Policy document search results

A total of 29 policy documents were identified and analysed to understand how they affect zoonotic disease management in India. The policy documents reviewed were mostly sectorally focussed, with 62% primarily on public health and 28% on animal health respectively (Table 3). Three international policies namely International Health Regulations 2007, OIE Terrestrial Animal Health code and the WTO Agreement on the Application of Sanitary and Phytosanitary Measures (SPS) Agreements were also reviewed as they were of relevance to the broader context of zoonoses management in India. The full list of policy documents included in the analysis is appended as Additional file 2.

**Table 3:** Key policies affecting zoonoses management in India (*n* = 29)

Domain	n (%)
Does this policy emphasise collaboration with all other relevant government sectors?	25 (86)
Does this policy emphasise collaboration with all relevant non-governmental organisations operating within public/ animal health interface?	18 (62)
Does this policy have a primary focus on public health?	18 (62)
Does this policy have a significant focus on animal health?	8 (39)
Does this policy have a significant focus on zoonotic diseases?	12 (41)
Can any zoonotic disease or its key drivers be clearly identified in conjunction with this policy?	8 (28)
Is this policy coherent with the wider One-Health framework?	5 (17)
Are there adequate/clear measures in place to ensure the sustainability of the programme/ policy?	4 (14)

### Low policy visibility of zoonotic diseases in India

To the extent that the policy context of zoonotic diseases has far-reaching implications for cross-sector control and management, it was instructive to examine their prioritisation across key sectoral policies as shown in Tables 3, 4 and 5. Tables 3 and 4 highlight two important points. First, the policy review found that zoonotic diseases tend to have very limited visibility or expression in the existing policy agenda setting, except for some mention in the National Livestock Policy (NLP) 2013, National Wildlife Action Plan (NWAP) 2017–2031 and the National Policy on Treatment of Rare Diseases (NPTRD) 2018. Of the 29 policy documents reviewed, only 12 (41%) had a significant focus on any zoonotic diseases of relevance to India. The National Livestock Policy (NLP) 2013 for instance, acknowledged the prevention, control and eradication of various disease conditions (including zoonoses) as critical to safeguarding livestock health but fails to spell out specific mechanisms or strategies on how this would be achieved. Interestingly, the recently promulgated National Health Policy (NHP) 2017, India’s flagship health policy (which replaced the NHP 2002), despite its ambitious and well-intentioned focus on adequate response to the changing health needs of India, the document fails to mention zoonoses as an important health concern and offers no guidelines on promoting cross-sectoral action; clarity on engagement with animal and/or forest sectors is conspicuously absent in the policy. This appears to give the indication that whilst zoonoses continue to gain currency in the international and national health discourses, they have yet to find sufficient expression even in relevant national sectoral policies [13, 30, 41]. Indeed, this observation was corroborated by a number of our interviewees who reported that the control of zoonotic diseases was not (yet) perceived as a shared responsibility or objective across the focal sector agencies, reinforced by the limited articulation of zoonoses in formal policy documents we reviewed (Table 3).

**Table 4:** Key sectoral policies affecting zoonoses management in India

Policy	Sector	Key focus
**National Health Policy (2017)**	Human Health	Seeks to stimulate innovation to meet health needs but silent on tackling zoonoses and no clear guidelines on cross-sectoral action.
**Draft National Pharmaceutical Policy (2017)**	Human Health	States that one of its key objectives is to create an enabling environment for R&D to produce innovator drugs, but silent on drugs or vaccines for zoonotic diseases. Policy yet to be operationalised.
**National Policy on Treatment of Rare Diseases (2018)**	Human Health	Underscores the importance of cross-sectoral approach to tackle rare diseases (including infectious diseases) but has not prioritised diseases and areas for research or how innovation will be supported.
**National Vaccine Policy (2011)**	Human Health	Focuses on strengthening R&D for the development of new vaccines to eradicate morbidity and mortality due to vaccine preventable diseases but does not mention vaccines for zoonotic diseases.
**National Livestock Policy (2013)**	Animal Health	States that one of its key objectives is to strengthen overall animal health through cross-sectoral action on prevention, control and eradication of various disease conditions (including zoonoses) but fails to spell out how this would be achieved.
**Science, Technology and Innovation Policy (2013)**	Human Health/ Animal Health	Underscores the need for multi-sectoral collaboration in India’s R&D system (including health and drug discovery) but does not spell out the specific contours of each sector and roles or how innovation will contribute to improved diagnostics and surveillance critical for prevention and control of zoonoses.
**National Wildlife Action Plan (2017–2031)**	Environment Health	Includes wildlife health as one of its thematic areas of focus and seeks build capacity of veterinarians of the State Animal Husbandry Department in forest bearing districts to tackle zoonoses but lacks clarity on how key actions will be operationalised. It also fails to outline how cross-sectoral coordination will be strengthened which is a key missing link in the plan.

**Table 5:** Policy visibility of important zoonotic diseases in India

Zoonotic disease	Status	Host involved	Existence of national programme		Notification status^a^	
			Human	Animal	Human	Animal
***Bacterial***						
Anthrax	Endemic	Livestock, Humans and Wildlife	×	×	×	√
Brucellosis	Endemic	Cattle, Buffalo, Sheep, Goat, Pigs and Humans	×	√	×	√
Tuberculosis	Endemic	Cattle, Humans	√	×	√	×
Leptospirosis	Re-emerging	Humans, Livestock and Rodents	√	×	√	×
Plague	Re-emerging	Rats, Cats, Humans	√	–	√	×
Scrub typhus	Re-emerging	Rodents, Humans	×	×	√	×
Salmonellosis	Re-emerging	Poultry, Livestock, Humans	×	×	×	×
***Viral***						
Avian Influenza	Emerging	Poultry, Ducks, Humans	×	√	×	√
Chikungunya	Re-emerging	Rodents, Humans,	×	×	√	×
Crimean-Congo Heamorrhagic Fever (CCHF)	Emerging	Livestock, Humans	×	×	√	×
Dengue Fever	Emerging	Monkeys, Humans	√**	×	√	×
Japanese Encephalitis	Re-emerging	Rodents, Livestock, Humans	√**	×	√	×
Kyasanur Forest Disease	Re-emerging	Rodents, Shrews, Monkeys, Humans	×	×	√	×
Nipah	Emerging	Livestock, Bats, Humans	×	×	√	×
Rabies	Endemic	Dogs, Bats, Humans	×	√	×	√
***Protozoan***						
Leishmaniasis	Endemic	Cats, Humans	√	×	√	×
Toxoplasmosis	Endemic	Cats, Ruminants, Humans	×	×	×	×
***Helminths***						
Cysticercosis	Endemic	Cattle, Pigs, Humans	×	×	×	×
Echinococcosis	Endemic	Dogs, Livestock, Humans	×	×	×	×

√ Denotes presence of a specific (own) national programme

√* Denotes presence of national programme in select states/ cities

√** Denotes presence of a joint control programme

× Denotes absence of national programme

Source: Authors modification based on Asokan et al. [8]

^a^Notification status of respective Diseases in under the IDSP/ NVBDCP (human health) and NADRS (animal health) reporting systems

However, comparing Tables 4 and 5 what emerges is that different zoonotic diseases have different recognition (visibility) on the disease prioritisation scale mediated by factors such as disease burden, species affected, morbidity and pandemic potential, international obligations etc. Inferring from Table 5, more policy attention seems to be on global zoonoses such as Plague, Avian Influenza (AI), Leishmaniasis, Leptospirosis, Brucellosis, whose human and economic impacts (and pandemic potential for AI and plague) have been well quantified. Less attention is given to endemic and re-emerging threats like scrub typhus and KFD that affect rural marginalised populations and whose burdens and impacts are probably significantly under-estimated. Second, Tables 3 and 4 also demonstrate the relative lack of multi-sectoral planning (in terms of operational frameworks that builds on the capacity of sectors for partnerships to occur) in some areas. Although some of the policies (*n* = 5, 17%) stated or described principles that were coherent with the wider ‘One Health’ ethos of advancing cross-sectoral collaboration within the public-animal health interface, there are very limited guidelines on how to foster, manage and sustain collaborations. For example, Section 13.7 of the NLP 2013 states that “One health” concept will be strengthened through cross-sectoral linkages with other concerned departments, such as Department of Health and Family Welfare. However, the mechanisms through which cross-sectoral coordination should be achieved were not clearly defined, and no corresponding government programmes had been introduced. Likewise, the NWAP (2017–2031) underscored inter-departmental collaboration between forestry and wildlife and animal husbandry departments in the areas of disease surveillance, monitoring and vaccination, but failed to clearly spell out how such partnerships should be operationalised on the ground to realise the intended outcomes. It therefore follows that part of the complexity and limited focus on zoonoses is rooted in the lack of supportive policies that create the enabling environment for galvanising cross-sectoral action. This inference is supported by the key informant interviews as participants asserted that despite the growing recognition of the cross-sector engagements to tackle zoonotic diseases, the lack of up-to-date integrative policy frameworks at multiple levels have constrained effective cross-sectoral action. A typical view in this regard is given by one interviewee as follows:

*“There is limited of cross-sector engagement at almost every level for almost every zoonosis. Because we don’t have a one health policy. The government has now started talking about it, and is now making the right noises but we still don’t have an integrative one health policy … ”* (Interview 3, Environment)

The discussion below draws on the key informant interviews on the barriers and facilitators to effective cross-sectoral collaboration for tackling zoonotic diseases in India.

### Barriers to effective cross-sectoral collaboration

Although participants generally considered the implementation of OH cross-sectoral approach to be critical for efficient disease surveillance and control, several cross-cutting barriers including: (1) technical (communication and information asymmetries and differences in disciplinary training), (2) institutional (institutional bureaucracy, perceived mistrust, ‘egos’, different mind-sets among actors, insufficient funding and coordination challenges), and (3) contextual factors (entrenched hierarchy and differences in regional capacities) were perceived to constrain collaboration in practice (Table 6).

**Table 6:** Barriers to effective cross-sectoral collaboration

Category	Barriers to integration
Technical	Limited infrastructure and resourcing
	Limited knowledge on zoonoses by relevant cross-sector actors
	Differences in training
	Disparate human and animal disease reporting systems
	Communication and information asymmetries
Institutional	Institutional bureaucracy and coordination challenges
	Competing departmental priorities
	Mistrust between actors, egos and different mind-sets among actors
	Different administrative cultures or working practices
	Insufficient funding allocation
	Disciplinary hierarchies and differences
Contextual	Complex disease governance system
	Entrenched hierarchical system
	Unsupportive policies
	Differences in regional capacities
	Regional/ cultural differences

#### Communication and information asymmetries

Participants from all sectors and all levels identified factors related to information asymmetries and effective communication as a major barrier to cross-sectoral engagement. The majority of interviewees described the lack of information on disease burden, incidence and transmission pathways particularly among human and animal health actors as constraining their potential contribution to disease control and prevention efforts. This was exemplified in the disparate human and animal disease surveillance and reporting systems, IDSP and NADRES which impeded effective data sharing between the two sectors. Indeed, a number of national and state level interviewees noted the need for integration of the two surveillance systems from the human and animal health sides as a critical step in filling identified knowledge gaps on zoonotic diseases. Yet, the perception that zoonoses was a human health issue (not (yet) a shared problem) among some non-health actors was seen to further complicate the relationship between sectors as there was the underlying expectation that the human health sector should spearhead efforts to address the related risk factors. This challenges implicit assumptions about the level of knowledge among cross-sector actors (especially stakeholders within the health sector) about the scale of the problem and their potential roles with respect to cross-sectoral collaboration efforts [21, 42]. It also echoes the underlying challenge of fostering support for sectoral ownership of One Health institutionalisation.

#### Perceived mistrust, “egos” and different mind-sets among actors

From the key informant interviews, mistrust (perceived or actual) between actors, differences in mind-sets and “deep-seated egos”, particularly around the leadership of cross-sector initiatives and how actors viewed each other were highlighted as key barriers to sustaining cross-sectoral action. From the perspective of public health actors, they perceived themselves to take a ‘supervisory lead’ in forging cross-sectoral partnerships as health needs take precedence over other sectoral imperatives. By contrast, actors in the animal health and environment sectors tended to view the public health actors as usurping or annexing “recognition” from cross-sectoral efforts and taking for granted or assuming that other sectors will necessarily contribute to their (public health) priorities. This perception was further accentuated by the common belief that each sector had its own mandate (“portfolio”) and that it does not necessarily have to implement the policies/ guidelines spearheaded by other sectors. The compartmentalisation of the human health and animal health departments under MoH&FW and MoA&FW respectively was perceived as key contributory factor in entrenching the bureaucratic competition that particularly existed between human and animal health departments which also affected the implementation of cross-sector initiatives. Indeed, human health actors were sometimes perceived to undermine their veterinary colleagues (as “playing second fiddle to medical doctors”) and did not fully appreciate their (veterinarians) potential contribution to disease control efforts. Animal health actors perceived this to inhibit the establishment of successful collaboration dynamics for cross-sectoral action:

*“Veterinarians in India have always felt somehow under-dogs as far as the medical profession is concerned. Many of them perhaps had wanted to become human doctors and couldn’t get through. So, there is underlying this feeling that medical profession is more specialised and know much more about something and veterinarians are peeved about something. And rather than working together complementing their strengths and knowledge system, they tend to be competitive. That I think is unfortunate … ”* (Interview 13, Animal Health).

These sensitivities were also recognised by some public health stakeholders as well:

*“I think this is the kind of socialisation in India. The medical profession is highly competitive, so people often think that those who can get admission into medical schools and eventually become (human) doctors have a higher social standing (considered as very senior members of the society) commanding lots of respect relative to those who train as animal doctors (veterinarians)...”* (Interview 9, Public Health)

Moreover, the forest and wildlife sector was in some instances perceived as a ‘fringe actor’ or ‘outsider’ with a marginal role (little) or no contribution in control efforts outside of specific outbreaks. Indeed, this rather implicit view was particularly telling among some public health actors, as a state level policymaker remarked: *“we (public health) are self-sufficient … it is up of the animal health guys to advance cross-sectoral collaboration. I don’t know much about the involvement of the wildlife in this case …*” (Interview 5, Public Health). In a unifying sense, the above feeds into a broader socio-cultural or class perception about the social status of animal doctors (veterinarians) relative to human doctors in contemporary Indian society as reflected by an Indian public health expert during an interview:

*“So most people are focused on either becoming doctors or engineers at the senior secondary level and obviously not everybody can get in - the system has way too many people coming in to take everybody in. For people who don’t get into the medical school maybe on first, second or third attempt, they tend to consider veterinary school as a fall-back option. This is not to say that veterinarians are less trained or less capable or medical physicians are more trained, it is just the way the system works. And given that there is so much social capital invested in the education of children and if you don't get into what you want obviously that kind of dynamics is likely to inform the subsequent interaction between human and animal health workers …”* (Interview 1, Public Health).

Whereas these implicit hierarchies occur within the wider domains of the social setting, they also find strong expression in the professional relationships of collaboration dynamics more so in a context where political and social sensitivities around careers are high. It therefore follows that the barriers to cross-sectoral collaboration are far-reaching, beyond the technical aspects often characterised in One Health discourses.

#### Institutional bureaucracy, entrenched hierarchy and coordination challenges

As evidenced in Tables 3 and 4, most of the key sectoral policies lacked clear formal guidelines or frameworks to support cross-sectoral action. This finding was further corroborated in the interviews as the majority of participants described that collaborations were mostly predicated on the professional goodwill and informal inter-personal relationships between respective cross-sector actors, with very limited formal incentives (such as compensation for time invested, capacity building and contribution to career advancement/ promotion) to collaborate. Several of the state and district level interviewees observed that the differences in priorities (roles) with respect to the operational conditions placed on them by the administrative authority created an (dis-)incentive system that sometimes created tensions and departmental mismatches regarding collaborations. For example, a district health officer in expressing his frustration about KFD management remarked:

“*Sir, one should come here and see, then only they come to know the problem. Everything is put on the health department only, then what to do? No staff, no finance. Staff themselves get irritated and feel enough with job. It is difficult sir* (Interview 14, Public Health).

Participants also highlighted that time constraints vis-à-vis the additional administrative burden (i.e. managing the coordination processes) imposed by such cross-sectoral efforts affected their willingness to collaborate with other sectors. For instance, some state and district-level actors reflected that they were extremely busy and overwhelmed by the competing demands to meet their core departmental obligations, making it difficult to simultaneously respond to collaboration requests from other departments. A district level health officer and state-level policymaker had this to say:

*“Managing both is difficult. Very difficult. Really difficult to manage both these full-time roles, especially when there is KFD outbreak it even more difficult to balance the responsibilities of both roles. In PHC, people (create) uproar if (I am) not there at the location as a doctor, and if I don’t come here, admin work that affects the whole taluka will be pending. So, when there is an outbreak it becomes even more difficult …”* (Interview 14, Public Health-Taluka level)

*“At the state level, it is actually very difficult to have [cross-sectoral] convergence meeting … not frequently. District level you can have it frequently. State level it requires time. Whereas at the district level the District Commissioner (DC) will be the chairperson convening such meetings, at the state level different secretaries are there, all of the equal cadres and also to have a [inter-departmental] meeting we have to get the concurrence of the chief secretary. And having a meeting with a lot of workload they are having, it is very difficult …”* (Interview 5, Public Health-State level)

This finding is not surprising to the extent that state government agencies are often reported to be grappling with human resource and logistical challenges [5, 13, 30]. Importantly however, some interviewees highlighted some state-based differences in terms of working cultures or practices which also affected (negatively or positively) the outcomes of cross-sectoral actions. As explained by one interviewee, a public health expert, whereas some states had a less formalised way of working, affording the space for collaborations to take-off organically and in a timely manner, others tended to have more formalised structure requiring more systematic overtures. A typical view of one interviewee is illustrative:

*“Different states have different cultures of working so a state like Tamil Nadu and I would expect state like Karnataka also would have a fairly formalised bureaucratic system whereas other states in India would have a strikingly less ad-hoc way of working. Some states would prefer having more formal committees in place to ensure collaborations, some states would prefer collaborations are done in a much informal way …”* (Interview 1, Public Health)

Other perspectives from local-level actors were that they could leverage on their social capital through informal interpersonal relationships and networks to circumvent existing departmental bureaucracy that hinders information exchange and cross-sectoral coordination efforts. As one interviewee explained, this was particularly evident during disease outbreak situations:

*“Let me give a little example from rabies. Almost any time the (human) doctor friends of mine come across a case of dog bite, they almost immediately call me to find out what the vaccination regime should be. So actually there is a lot of scope for veterinarians and doctors to work close together when the need arises, example during disease outbreaks …”* (Interview 13, Animal Health)

While these informal collaborative networks seemed to work well in some instances, some respondents expressed concerns that they could fail in the face of unanticipated circumstances (such as staff rotations or transfers, departmental leadership changes) due to the lack of explicit formal agreements between sectors. As one participant noted, *the lack clear guidelines within policy framework constrained the opportunities or incentives needed to sustain cross-sectoral coordination efforts outside of outbreak episodes* (Interview 7, Public Health). Consistent with the literature [13], a number of interviewees highlighted the failure to leverage on collaborations that arose during the relatively successful avian influenza control in 2008 as a missed opportunity to institutionalise One Health.

#### Insufficient funding allocation

Contrasting views between state and district level actors on resource allocation for zoonotic disease control highlight some incongruence which impacted significantly on collaboration dynamics. Almost all district level actors from the human and animal health sectors felt strongly that additional resources should be allocated to their respective departments or to zoonotic disease control. They further argued the need to set up dedicated funding streams to foster cross-sectoral actions, without which cross-sector partnerships are difficult to sustain, especially when cost is involved. Two typical views expressed by a taluka level animal health official and veterinarian when asked if there is enough funding for zoonotic disease control are illuminating:

*“No sir, funding, there is no funding for that purpose alone, we give whatever we have in the department, for example, they give fund of x lakh*^6^*in Zila Panchayat*^7^*(District Council) to our department, so, there are 7 talukas, it comes approximately xx lakh for each taluka. So, in that y lakh, deworming, antibiotic, dressings and all, everything we should give. Sir, I have 29 to 30 hospitals, it is more if yy lakh comes for one hospital.”* (Interview 15, Animal Health- Taluka level)

*“Ah! Government funding to me remains a mystery [laughing]. We are still struggling to access some government funding for a project since last year (2018). We always hear there is money but we don’t see it. Perhaps it is the way the system is structured and bureaucracy issues...”* (Interview 12, Animal Health)

Yet national and state level respondents, particularly from the human health sector did not perceive funding to be a challenge. In fact, all of the national and state level actors argued that resources for disease control were available, making references to dedicated funding under the centrally-sponsored nation vector-borne disease control programme (NVBDCP) and the integrated disease surveillance project (IDSP). To buttress this assertion, one state level respondent intimated that there was significant amount of unused funding allocation for the financial year and that what is actually needed is the reorganisation of budgets (budgetary planning) by respective departments to enable more efficient use of allocated resources. A state level programme officer had this to say:

*Funding is not a problem nowadays. After National Health Mission we have got a lot of funds. So, funding is not a problem for any activity. It is only the mind-set and commitment that is required from the employees …”* (Interview 5, Public Health)

The conflicting views of top-level bureaucrats and district level implementers on resource allocation highlights a seeming disconnect between policymakers and implementers on the ground. A number of reasons could explain this contradiction. First, as some interviewees disclosed, the existing bureaucratic setup sometimes contributed to delays in the disbursement of centralised or state level funds, resulting in some departments been starved of funding for designated activities. Second, the political sensitivities around the central government funding has meant that senior state-level policy makers sometimes shy away from openly complaining about funding or other constraints they are facing, as doing so has the propensity to negatively affect one’s position and their overall departmental outlook. Indeed, a senior state-level bureaucrat implicitly echoed this sentiment suggesting that *“the top-level people are in a better position to speak about these challenges …”* (Interview 10, Public Health).

#### Differences in disciplinary training

Differences in the disciplinary training was discussed as key underlying barrier to fostering cross-sectoral action. Participants intimated that the disparate training of veterinary and medical professionals, despite the many commonalities, contributed to the ‘silo-based’ thinking of professionals within their respective disciplines. A typical view in this regard was given by a public health expert when reflecting on of the health pedagogy in India:

*“So veterinary public health is very similar to veterinary microbiology because they both tend to be very lab-based focussed and pathogen-based focussed except that veterinary public health they do focus on zoonotic pathogens and veterinary microbiology non-zoonotic pathogens affecting animals. This is quite a contrast to the way public health graduates are trained in human health medicine. The public health training is still much more sensitised to epidemiology, burdens from a human health point of view... So there’s limited overlap in the training which also affects subsequent interaction between human and animal health workers”* (Interview 1, Public Health)

Some interviewees argued that an integrative OH pedagogy could contribute positively towards professionals “thinking outside their disciplinary boxes”, more collaboratively and instilling a sense of mutual trust and respect. This observation also dovetailed with concerns about cross-disciplinary approaches to alleviate information asymmetries and entrenched professional hierarchies:

*“How to create that kind of environment where medical and veterinary professionals or one health people from different disciplines come together and have mutual respect is a big challenge. This perhaps requires some trans-disciplinary modules in the curriculum that exposes students to the value of inter-discliplinarity which could be a useful starting point …”* (Interview 9, Public Health)

It therefore follows that effective cross-sectoral action transcends technical “fixes” to other structural issues in the education and training of professionals. As McKenzie et al. [24] argue joint education for professionals from different sectors serves as an effective means of building understanding and trust between disciplines and breaking down the barriers and misconceptions between the medical and veterinary professions. Professional hierarchies notwithstanding, collaborations shaped around outbreak responses suggest a different picture on working across sectors. As a key informant highlighted, *during outbreak events, veterinarians and medical professionals are able to work together and mutual respect and trust develops in the process* (Interview 5, Public Health). This underlines the view that short-term collaborations in the event of outbreaks are more likely than long-term systematic collaborations which require formalised understanding between disparate institutional bureaucracies.

### Facilitators of effective cross-sectoral collaboration

Aside from identifying barriers, participants provided suggestions to address the aforementioned challenges and facilitate cross-sectoral convergence (see Table 7). From our analysis, four main sub-themes viz. (1) governance mechanisms, (2) clear delineation of sectoral roles, (3) communication and formalised relationships, and (4) resourcing arrangements, provide a good window of opportunity or critical entry points for strengthening cross-sectoral convergence.

**Table 7:** Facilitators of effective cross-sectoral collaboration

Category	Facilitators of integration
Formal governance and leadership structures	Existence of a National Standing Committee on Zoonoses.
	Full operationalisation of existing memorandum of understanding between ICAR and ICMR on zoonotic diseases.
	High-level political support/ commitment
Clear delineation of sectoral roles	Assignment of responsibilities/ roles defining for each sector/ department – e.g. Action Plan on Avian Influenza which has a clear delineation of the respective sectoral/ departmental roles and responsibilities for effective prevention and control of avian influenza.
	Cross-sectoral relationships based on shared understanding of the problem.
	Developing clear operational guidelines and frameworks for cross-sectoral collaboration.
Improving communication and working relationships	Building joint communication platform for data sharing on zoonoses based on existing human (IDPS) and animal disease (NADRES) reporting systems.
	Leverage on past and on-going collaborative mechanisms – e.g. Action Plan on Avian Influenza involving all relevant sectors/ departments for effective prevention and control of Avian Influenza.
	Building on existing informal networks/ mechanisms.
Resourcing considerations	Sectoral national and state funding for disease control and surveillance programmes in human and animal health.
	Regularisation of capacity building programmes in the areas of diagnostics and joint outbreak investigations – e.g. KFD technical training and capacity building on outbreak investigations.
	Technical support from international agencies and partners.

#### Formal governance and leadership structure

Participants commonly cited the establishment of formal governance mechanism as a prerequisite for successful operationalisation of One Health cross-sectoral approach. Whereas participants concurred on the need for a formal governance body which transcends the health sector to other focal non-health agencies, perceptions of who should lead this arrangement varied. Some key informants argued that OH requires a special secretariat at the national level, which could act as an ‘impartial agency’ that sits between sectors:

*“A central coordinating agency which would have people from the forest, public health, and animal health departments all deputed to this organisation which coordinates cross-sectoral activities. Such a mechanism affords opportunity for formal reviews, policymaking and formal accountability …”* (Interview 3, Environment)

*“My sense is that it should start from the top level because in our kind of countries, people generally look up to the seniors and at the top level if there are committees, one health committee, standing committees not only for epidemics but standing committees which means permanent committees which meet at some regular intervals - six monthly, annually or whatever and so they make policy and define roles for each of the departments for execution and then reconnect the review …”* (Interview 9, Public Health)

Others expressed the importance of building on the existing National Standing Committee on Zoonoses (NSCZ), which in their view, has the ‘high-level political support’ and a clearly defined mandate for action beyond the heath sector as specific entry point for operationalising a OH cross-sectoral approach. A few participants however expressed that institution of leadership structures at all levels would be a better way to ensure legitimacy and proper accountability. Two typical views in this regard expressed by an Indian public health expert and a veterinarian are as follows:

“*We have to recognise that cross-sector collaborations might take place at different platforms, in different places, at different levels and different kinds of issues. Standardised one-size-fits-all approaches to One Health in a formalised environment might or might not work in specific settings. I think if you have to have a better understanding of the development of different sectors you need to encourage multiple levels of interaction and in different ways for different functions. So allowing for flexibility within bureaucratic systems might be a good start …”* (Interview 1, Public Health).

*“In the case of a large country like India, south India, north, east, west … very different administrative culture, very different culture. So one kind of thing may not work everywhere …”* (Interview 12, Animal Health)

Although the varied perspectives connotes some disagreements, which constitute barriers themselves, there was a general understanding that without such a governance arrangement then the barriers to cross-sectoral action remain unsurmountable.

#### Clear delineation of sectoral roles

A common consensus among participants was that key sectoral roles and responsibilities needs to be well defined. Considering the different sectoral priorities, participants highlighted the importance of role clarification, especially for the non-health departments to incentivise their participation or time investment in cross-sector collaborations. The establishment of appropriate communication and accountability structures was also deemed necessary to afford better information exchange between sectors. In so doing, several participants also highlighted the need for a focal person at different levels to coordinate collaboration between sectors.

*“Creating operationalisation guidelines is necessary. This should be available in all the relevant departments and specify the respective roles in disease control efforts as these things may not be a priority in other (non-health) departments so they may not know what their roles are, what their responsibilities are. I think that kind of thing will help technically …”* (Interview 9, Public Health)

*“Rather than saying for One Health this department will take lead. Like they can decide for brucellosis this department has a major role so they will take lead but we will do this much. These things should be clearly specified that who will do that. And then budgetary allocation should be there for that, staff should also be there. And then accountability should also be there - say annually or six monthly each department should come together and say what they have done …”* (Interview 12, Animal Health)

#### Improving communication and working relationships

Several participants highlighted the integration of the disparate human and animal disease surveillance systems as a potential avenue to promote efficient information sharing between sectors. They also identified leveraging on existing networks as critical to bridging the communication gap between sectors.

##### Building on past collaborations and informal interpersonal relationships

Participants frequently reported that despite the limits imposed by professional and institutional hierarchies, cross-sector collaborations were happening especially during outbreak situations, albeit in less formal and ad-hoc way. The majority of interviewees thus underscored the need to leverage on existing networks and informal inter-personal relationships to initiate and sustain collaborative arrangements. Towards this end, some participants suggested memoranda of understanding (MOUs) as not only providing a formal basis for existing working relationships, but also recognising the contributions to cross-sectoral relationships, particularly the animal and environment health sectors. A state-level programme manager had this to say:

*“Whenever there is some outbreak or they (veterinary department) conduct some workshop we go there, then there is some interaction. It happens like that only but then we realised we need to meet more and more formally and we need exchange of information. This can be achieved through having champions and formal understanding to define the basis of the engagement and information sharing”* (Interview 4, Public Health)

##### Civil society and international partnerships

Participants indicated the engagement of civil society and international partnerships affords a useful convening agency to increase legitimacy and support for cross-sectoral actions. Some interviewees pointed to on-going collaborations between the US Centre for Disease Control CDC and the National Centre for Disease Control, RCZI and other project-based collaborations (e.g. the UK Global Challenge Research Funded Monkeyfeverrisk project) as creating critical platforms for cross-sectoral engagements, which provide wider perspectives towards strengthening OH collaborations.

#### Resourcing considerations

Aside from dedicated budgetary allocation for cross-sectoral activities, participants highlighted the need to develop infrastructure that advances OH interdisciplinary engagement. As one veterinarian argued, effective zoonoses management is not only contingent on working across sectoral boundaries, but ensuring that key actors have the institutional support and capabilities to collaborate with partners operating under different departmental norms. Other participants alluded to this factor observation underscoring that it is an important consideration, the absence of which could lead to a breakdown in cross-sector communication:

*“In some PHCs, we require technicians, we are adjusting and doing it now by deploying and moving people here and there depending on outbreak areas and need, but this is only a short-term measure. In the long term, all positions have to be filled. It should be streamlined and there should be protocol …”* (Interview 14, Public Health-District level)

*“Capacity building is very important. We have to train our people. Especially field level workers, programme implementers and planners. They have to be trained with respect to zoonotic diseases and One Health. That is very important. It has to be done regularly. At least quarterly or at least twice a year …”* (Interview 6, Public Health-State level)

## Discussion

**Fig. 3:**
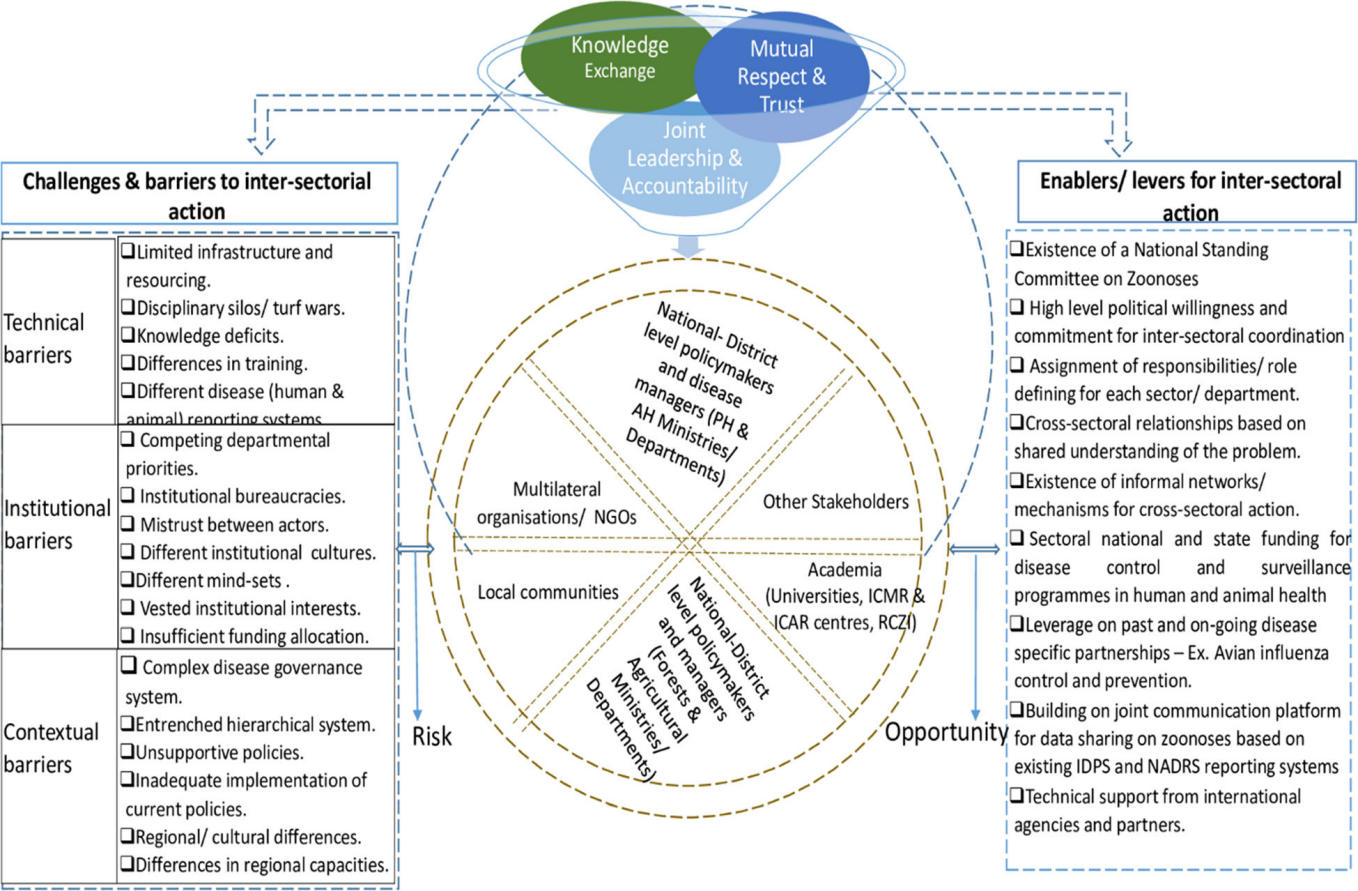
Building resilient multi-sectoral partnerships for zoonoses management. Structural characterisation of the key drivers of cross-sectoral collaboration and actors in India’s zoonotic disease system. AH = animal health; PH = public health; ICMR = Indian Council for Medical Research; ICAR = Indian Council for Agricultural Research; RCZI = Roadmap to combat zoonoses in India

There is widespread consensus that achieving cross-sectoral collaboration between health sector and non-health sectors is challenging [14, 16, 33]. In our study, we explored the national policy context and conditions for cross-sectoral convergence between human health, animal health and environment sectors as a pathway to improved disease surveillance and control in India. As noted in the wider literature, which our findings support, effective multi-sectoral convergence is contingent on a host of interacting factors and mechanisms for improved cross-sectoral actions and positive outcomes [5, 14, 30, 33]. It therefore follows that developing resilient and sustainable cross-sectoral partnerships in practice is on the one hand, predicated on overcoming the barriers that hinder collaboration, and on the other hand, leveraging on the existing opportunities or factors that enable and facilitate cross-sectoral action (see Fig. 3).

The findings of this study showed widespread acknowledgement of the importance of multi-sectoral convergence for controlling zoonotic diseases among participants. Almost all participants highlighted the value of cross-sector partnerships in affording a more holistic understanding of zoonoses and improved surveillance across the human-animal-environment interface. This reflects that a common understanding of the need for cross-sectoral engagement is present at different levels, but key remaining barriers need to be addressed in order to enhance cross-sectoral collaboration. Although 86% of national policy and program documents from the health and environment sectors demonstrate a common recognition of the importance of multi-sector convergence, there are gaps in provision of operational guidelines necessary to implement cross-sectoral action (see Tables 3 and 4). Related to this is the low policy visibility of zoonoses in the existing policy agenda setting and the varying levels of prioritisation of specific diseases within policy. The low policy visibility of zoonoses translates into limited awareness and resource investment, which could potentially hamper the development of operational guidelines for effectively addressing them. To the extent that this inference holds true, then it is not entirely surprising the conclusion by some analysts [25] that India’s response to zoonotic diseases has at best been ad-hoc and altogether reactive notwithstanding the considerable success in disease control.^8^ This echoes the need for sustainable cross-cutting national policy frameworks to underpin disease interventions.

Significantly, the findings of this study reveal important facilitators of and barriers to successful collaboration across sectors (Tables 6 and 7). Whilst consistent with the literature [7, 13, 23, 43], we however identified that some facilitators and barriers are more important than others which the Indian authorities (in collaboration with international partners) need to consider and prioritise in operationalising OH to engender the requisite buy-in across the different sectors and improve collaborative outcomes. Within this purview, our findings suggest that the evolving socio-political context operates to significantly shape how enablers and barriers are perceived. For instance, the hierarchical and bureaucratic setup of the health system coalesced with competing departmental priorities and “egos” to entrench existing sectoral “silos” and limit the ‘spaces’ for formal collaboration between health and non-health sectors. Since these largely socio-political factors shape collaboration dynamics in practice, the narrow framings of OH cross-sectoral collaboration as a technical issue fail to recognise the interplay of contextual factors within national and local socio-political systems that determine collaborative outcomes [30, 44].

Consistent with the literature, our findings suggest that without sufficient consideration of important contextual factors, a wholesale or ‘one-size-fits-all’ implementation of One Health approaches in could at best be ineffective and at worst further exacerbate the very challenges they are meant to resolve [14–16, 30]. Within this purview, our results indicate that knowledge and effective leadership are important factors in facilitating collaborations and alleviating the challenges to cross-sectoral convergence. As evidenced above, participants frequently reported that effective leadership, exemplified through formal and informal working relationships at different levels, was instrumental in bridging the communication gaps that impeded effective cross-sectoral action. This reflects the notion that cascading cross-sectoral structures from the central to local levels will require improved awareness, leadership and sustained political commitment [13, 15]. Although strengthening channels of communication among policymakers, disease control programme managers, and other sectoral actors is important, that alone is unlikely to sufficient and cross-sector advocacy may be required to sustain commitment and to improve cohesion and coordination [12]. Within this purview, the identification of champions (within the program implementation system and research arena) who are empowered to advocate for cross-sector engagement at different levels may be necessary, particularly to ensure that such engagement remains a priority and to support or inspire the orientation of staff from relevant non-health sectors, that have traditionally operated at the fringes of zoonotic disease intervention programs [4, 45]. In doing so, the need for the establishment of financial and non-financial incentives (such as formal acknowledgement or recognition and continuing professional development opportunities), and accountability systems to stimulate cross-sectoral engagement as shown in this study cannot be overemphasised [12].

Our findings also highlight that successful cross-sectoral collaboration is largely rooted in mutual trust and respect between sectoral actors. Participants commonly reported that collaborations are less likely to be open in sharing sensitive information and tacit knowledge if a certain level of trust does not exist between departments. As evidenced in the interviews, mistrust between departments (perceived and actual) limits opportunities for information sharing among actors. Virani [46] and Kogut and Zander [47] have argued that trust-based relationships can facilitate the exchange of different levels of knowledge and information. Equally important are funding and infrastructural resourcing which serve as a catalyst for the formation and sustenance of cross-sectoral action. As participants reported, the limited capacities of respective departments has meant that they had very limited incentives to collaborate, particularly if intended collaborations sit outside of their core departmental mandates. This finding echoes Bogich et al.’s [48] argument that it is difficult to sustain collaborations without minimum capacities among all sectors. It may well also explain the basis of Chatterjee et al.’s [26] argument that south Asian countries (including India) lag behind those in the Southeast Asian region regarding institutional capacity for OH research and response. The allocation of pooled funding for cross-sectoral activities and adequate transfer of resources to relevant departments, particularly at the district levels, as suggested by participants, remain critical to engendering the appropriate incentives needed to galvanise widespread support for cross-sectoral action. This derives from Craddock’s [33] observation that collaborations become more productive only when particular interests converge and when larger financial and political conditions fall in line with collaboration ambitions. Indeed, interviewees commonly intimated that the huge policy and public panic following the outbreak of H5N1 Avian Influenza in India, for example, triggered some ‘interest convergence’ as the human health, animal health and wildlife sectors collaborated to successfully address the problem. This collaboration culminated in the establishment of an Inter-Ministerial Task Force (IMTF) and Joint Monitoring Group (JMG) at the national level, with decentralised coordination mechanisms and operational guidelines down to the district level [13, 49, 50].

Based on the findings from this study, a flexible contextual OH adaptation (involving a mixture of both top-down and bottom-up governance) that accommodates the complexity and diversity of interests is recommended to overcome the barriers and leverage on existing opportunities for developing resilient OH cross-sectoral collaborations. This follows from the fact that developing and sustaining cross-sectoral collaborations is not straightforward but highly complex and predicated on several context-specific factors that need critical consideration. Indeed, past experience with the NCDC-led centralised platforms (e.g. the National Standing Committee on Zoonoses (NSCZ)) offer no basis for thinking that centralised governance mechanisms alone would do a better job than multi-level platforms, involving several actors operating at the human-animal-environment interface of zoonoses management. This is not to deny the legitimate mandate/ interest of centralised institutional frameworks considering the political capital and technical capacity required to complement state-level capacities in disease surveillance and control. In any event, the establishment of centralised OH structures for zoonoses control “whilst desirable as a politically endorsed “glue” to hold everything together, will ultimately need to weather the inter-ministerial “turf-wars” likely to emerge as a result of ministries attempting to maintain control over resources and policy arenas” ([16], 266–267). Equally important is the fact that the institutional/ administrative expediency pertaining to institutionalising OH varies from state to state, which underscores potentially large regional (state-based) differences that can affect cross-sectoral collaborations. In these circumstances, flexible multi-level OH platforms such as Kenya’s sub-national County One Health units (CoHUs) responsible for coordinating cross-sector disease surveillance and outbreak response activities at the county and sub-county levels, hold the greatest promise [51]. Whilst the Kenyan model is not without shortcomings, particularly at the sub-national level, it seems to reflect that decentralised OH governance affords the flexibility of accommodating importance regional differences (in resourcing and working practices) for cross-sectoral action, as in the case of India. Learning from such experiences to inform OH operationalisation in India is recommended as a wholly centralised or standardised approach to OH implementation will do very little to address the underlying barriers to effective cross-sectoral action for zoonotic disease prevention and control. As Lee and Brumme ([15], 780) argue, “simply grafting OH onto the existing institutional structures is likely to pose risks”. This indicates a requirement to think more deeply about the implementation of OH cross-sectoral approaches, particularly in developing contexts like India.

This study is not without limitations. First, as we reviewed only the main policy and program documents affecting zoonotic disease management, we may have missed information contained in supplementary documents, such as those enacted at the state government level, and may therefore have understated focal policies. Second, although our approach was to ensure that policies we reviewed were current at the time we obtained them, we cannot dismiss the possibility that we missed a newer document or that a policy was updated before this review was completed. Third, we also acknowledge that the small and disproportionate sample across sectors is a limitation of this study, given that environment and animal health sectors play a profound role in OH cross-sector convergence for zoonotic disease control. Nevertheless, the broad coverage of existing national policies and programmes, enabled by the careful selection of key informants, afforded in-depth qualitative insights into the current state of play on zoonotic disease control and multi-sector partnerships within the Indian health systems. Finally, the timing of the current study vis-à-vis the emergence of the COVID-19 pandemic presents a potential avenue to alter policy and institutional relationships and information systems, necessitating further examination over time.

## Conclusions

The present study adds to the existing body of knowledge regarding national policy frameworks for zoonoses prevention and control and the conditions for advancing successful cross-sectoral collaboration across the human-animal- environment interface in India. This is against the backdrop of the clarion call for widespread adoption of OH cross-sectoral approach for achieving better health and non-health outcomes [3, 12, 25, 52]. As demonstrated in this study, there is limited policy visibility on zoonotic diseases particularly high burden endemic diseases that disproportionately affect marginalised rural populations in India. Although the existing policies acknowledged the importance of cross-sectoral action for effective zoonoses control, they generally lacked clarity on guidelines or actionable measures for achieving such engagement in practice. Our findings suggest that beyond the institution of favourable policy frameworks, achieving multi-sectoral convergence is not linear but complex and multi-layered, involving mediators in different positions in-between and at the intersection of these hierarchical structures. Within this purview, existing networks and inter-personal relationships, strong leadership, trust and accountability, knowledge and policy frameworks are important facilitators to and/ or barriers to cross-sectoral action. Our findings therefore highlight the importance of clarifying or strengthening national policy frameworks (through critical assessment of policy gaps) as an important first step for leveraging cross-sectoral capacity for improved disease surveillance and interventions. This further imposes a requirement for the contextual adaptation of the OH approach in a manner that is sensitive to the underlying socio-political and cultural context that determines and shapes outcomes of cross-sector collaborative arrangements.

## Supplementary Information

**Additional file 1.** Interview Guide for Key Informants.

**Additional file 2.** List of policy documents included in final analysis.

**Additional file 3: Supplementary Figure 1.** Schematic representation of the organisation of the human health sector in India, showing the network of actors and information flow. Source: Planning Commission of India (2011) as cited by Gupta and Bhatia (undated). The Indian Health Care System. Available online at https://international.commonwealthfund.org/countries/india/ (Accessed on 19/02/2020).

**Additional file 4: Supplementary Figure 2.** Schematic representation of the organisation of the animal health sector in India, showing the network of actors and information flow. Source: Authors’ construct.

**Additional file 5: Supplementary Figure 3.** Flowchart of the selection process of relevant documents. Authors’ construct.

## Data Availability

Given the richness of the qualitative data and the potential for identifying individual human participants and violating confidentiality of the key informants who participated in the semi-structured interviews, our study dataset will not be shared openly but may be available upon specific request. Researchers wishing to access the dataset used in this study should contact the UK Centre for Ecology & Hydrology Institutional Data Access contact via Jim Chiazzese (email: jimchi@ceh.ac.uk) .

## Abbreviations

ASHA: Accredited Social Health Activist
CDC: US Center for Disease Control
DAHD: Animal Husbandry Department
FAO: Food and Agricultural Organisation
GOI: Government of India
HPAI: Highly Pathogenic Avian Influenza
ICAR: Indian Council for Agricultural Research
ICMR: Indian Council for Medical Research
IDSP: Integrated Disease Surveillance Programme
IMTF: Inter-Ministerial Task Force
JMG: Joint Monitoring Group
KFD: Kyasanur Forest Disease
LMIC: Low/Lower Middle Income Country
WHO: World Health Organisation
ANM: Auxiliary Nurse Midwife
MoH&FW: Ministry of Health and Family Welfare
MoA&FW: Ministry of Agriculture and Farmers Welfare
MoEF&C: Ministry of Environment, Forests and Climate Change
NADRES: National Animal Disease Referral Expert System
NCDC: National Centre for Disease Control
NGO: Non-governmental Organisation
NLM: National Livestock Mission
NSCZ: National Standing Committee on Zoonoses
NVBDCP: National Vector Borne Disease Control Programme
OH: One Health
OIE: Office International des Epizooties (World Animal Health Organisation)
RCZI: Roadmap to Combat Zoonoses in India
R&D: Research and Development
SARS: Severe Acute Respiratory Syndrome
SDG: Sustainable Development Goals
UN-REDD: United Nations Reducing Emissions from Deforestation and Forest Degradation Programme
VCI: Veterinary Council of India

## Footnotes

1 According to Kahn [1] One Health is defined as a worldwide strategy for expanding interdisciplinary collaborations and communications in all aspects of health care for humans, animals and the environment.

2 In this paper, cross-sectoral convergence is defined as people and organisations from multiple sectors working together for a common purpose following after Rouse and Fawcett [4]. Importantly, in the literature collaboration or convergence is widely viewed as a continuum ranging from disparate functioning to sharing information (cooperation), to undertaking common activities (coordination) and merging resources and infrastructure (integration). In this view, terms such as integration, collaboration, coordination and cooperation are often used interchangeably or defined differently to depict stages along a continuum [5, 6]. We therefore use the terms ‘convergence’ or ‘collaboration’ in a generic fashion and as being synonymous with the overall continuum of integration [5, 6].

3 Prospective participants for the key informant interviews were identified based on the prior networks of the authors (BVP, SLH, MMC and ATV) and reference listings in scientific publications.

4 Divided into 29 states and 7 union territories, which have a level of administrative and legal autonomy.

5 The NCDC is the technical arm of the MoH&FW assisting the ministerial heads to determine national health policies through an interactive process using information and research evidence. Within the NCDC set-up is the National Standing Committee on Zoonoses (NSCZ) and the National Vector Borne Disease Control Programme (NVBDCP), which focuses on the prevention and control of vector borne diseases.

6 Lakh is a unit in the Indian numbering system equivalent to one hundred thousand.

7 Zila Panchayat is the third tier of the decentralised district-level governance or the Panchayat Raj system.

8 Successful control of avian influenza, the IDSP and NADRS information systems portal for disease surveillance
