# Supplementary material for: Operationalising the “One Health” approach in India: facilitators of and barriers to effective cross-sector convergence for zoonoses prevention and control
Source: BMC Public Health. 2021 Aug 6;21:1517. doi: 10.1186/s12889-021-11545-7 (PMC8342985; doi:10.1186/s12889-021-11545-7)
Supplement: Supplementary file 1 — Additional file 1. Interview Guide for Key Informants. [file 12889_2021_11545_MOESM1_ESM.docx]

**Additional File 1: Interview Guide for Key Informants**

**Introduction**

**We need a PIS and consent form before the interview.**

Thank you for your time for this interview. I have a few questions about the key policies affecting zoonotic diseases and disease management. Given your work and experience, we believe that it is important to understand better how to advance inter-sectoral collaboration within the human-animal-environment interface for improved disease surveillance and interventions in the country (or your state).

**General Information about respondent**

1. Details about the person, their job title, education background, place of posting, geographical area/scale over which they operate
2. Could you tell us about your experience and role with regards to zoonotic diseases and disease management? [***Probes****: role and years of involvement*]

**Key policies affecting zoonotic diseases and disease management**

1. Could you please share your thoughts about the main infectious diseases in your area?
2. Is there a formal prioritisation for zoonotic diseases in India?
3. If so, could you indicate whether you are in agreement with this prioritisation? I.e. is the current prioritisation justified? [***Probe****: RCZI*] If not, could you explain why?
4. If so, what factors informed the prioritisation?
5. Who carries out the prioritisation?
6. How often is the prioritisation refreshed?
7. Can you tell us about any policy/ program (current/ past) initiatives that you aware of to regulate and control zoonotic diseases? [***Probes:*** *Names of policy initiatives or control programs, origin (national/state/district) and salient features, level of implementation, and how they affect/ impact on your ability to manage identified zoonoses*]
8. What triggers the development of different programme/ policy initiatives?
9. In your opinion, are there international policies relating to zoonoses and animal health that affect India's health policies? If so, which are they?
10. In your opinion, are these policies/ programs initiatives effective in controlling the identified zoonotic diseases? [**Probe:** policy/ program relevance, *challenges in implementation – fragmented nature of policies/ programs, policy/ program gaps and level these are manifested*]
11. If so, why are they effective? If not, why are they not effective?
12. In your opinion, which other departments are critical for the successful implementation of policies/ programs relating to zoonotic disease management? [**Probe:** *different administrative levels departments should interact for effective* coordination, *frequency of interaction*]
13. Are there any specific policies/ strategies from other sectors that need to be taken into consideration when planning in the context of zoonotic diseases?

**Current management of zoonotic diseases and One Health Linkages**

1. Is there adequate consideration of cross-sectoral components in policy design? If so, how?
2. If not, what is missing? Which sectors need to be linked? Are there any specific policies/ strategies from other sectors that need to be included in the design phase of policies?
3. Is there adequate consideration of cross-sectoral components in policy implementation?
4. If not, what is missing? Which sectors need to be linked? Are there any specific policies/ strategies from other sectors that need to be included in the implementation phase of policies?
5. In terms of management of zoonoses, what are the major challenges that you face?
6. In your view, would you say policy alignment with One Health approach is a challenge with regards to zoonotic disease management? If so, in what way?
7. In your opinion, would you say inter-sectoral collaboration is a challenge with regards to zoonotic disease management? If so, in what way? Please list the top 2 challenges
8. In your opinion, would you say communication/ information asymmetries is a challenge with regards to zoonotic disease management? If so, in what way?
9. In your opinion, would you say institutional/ departmental deficiencies constitute a key challenge with regards to zoonotic disease management? If so, in what way?
10. In your view, to what extent does funding impact on the development, sustainability and implementation of zoonotic disease control programs? [***Probe:*** *different funding streams and how they impact on disease control arrangements*]
11. Based on your experience do you have any suggestions to improve the management of zoonotic diseases in your state?
12. In your view, are there better ways of addressing the policy alignment with One Health approach? If so, how?
13. Are there better ways of strengthening collaboration between the human and animal health sectors in their responses to disease outbreaks? If so, how?
14. Are there better ways of improving communication/ information flow between relevant departments/ institutions regarding zoonotic disease management? If so, how?
15. In your opinion, is an integrative One Health Policy necessary for zoonotic disease management in India? If so, why?
16. Is there any other management strategy that could contribute towards effective zoonotic disease management? If so, please explain:
17. Is there anything we have not covered that you want to add?

Many thanks for your participation in our study.
