# Supplementary material for: Operationalising the “One Health” approach in India: facilitators of and barriers to effective cross-sector convergence for zoonoses prevention and control
Source: BMC Public Health. 2021 Aug 6;21:1517. doi: 10.1186/s12889-021-11545-7 (PMC8342985; doi:10.1186/s12889-021-11545-7)
Supplement: Supplementary file 2 — Additional file 2. List of policy documents included in final analysis. [file 12889_2021_11545_MOESM2_ESM.docx]

**Additional File 2: List of policy documents included in final analysis**

1. National Health Policy 2017
2. Draft National Pharmaceutical Policy 2017
3. National Policy on Treatment of Rare Diseases 2018
4. National Vaccine Policy 2011
5. National Livestock Policy 2013
6. The Science, Technology and Innovation Policy 2013
7. National Wildlife Action Plan 2017 – 2031
8. The Epidemic Disease Act 1897
9. National Policy for Farmers 2007
10. National Agricultural Policy 2000
11. National Forest Policy 1988
12. National Environment Policy 2006
13. National Action Plan on Avian Influenza (Action Plan of Animal Husbandry for Preparedness, Control and Containment of Avian Influenza) 2005
14. The Revised National Tuberculosis Control Programme
15. National Roadmap for Kala-azar Elimination in India
16. Programme for the Prevention and Control of Leptospirosis
17. National Health Mission of India
18. National Vector Borne disease control programme (NVBDCP)
19. Integrated Disease Surveillance Programme (IDSP)
20. National Project on Rinderpest Surveillance & Monitoring (formerly National Project on Rinderpest Eradication (NPRE))
21. National Strategic Plan for Malaria Elimination in India 2017-2022
22. National Rabies Control Programme
23. Foot and Mouth Disease Control Programme (FMD-CP)
24. National Control Programme on Brucellosis (NCPB)
25. National Policy for Containment of Antimicrobial Resistance
26. Zero by 30: The Global Strategic Plan to Prevent Human Deaths from Dog-Transmitted Rabies by 2030.
27. WTO Agreement on the Application of Sanitary and Phytosanitary Measures (SPS Agreement)
28. OIE Terrestrial Animal Health Code (Terrestrial Code)
29. International Health Regulations (2005)
