# Supplementary material for: Operationalising the “One Health” approach in India: facilitators of and barriers to effective cross-sector convergence for zoonoses prevention and control
Source: BMC Public Health. 2021 Aug 6;21:1517. doi: 10.1186/s12889-021-11545-7 (PMC8342985; doi:10.1186/s12889-021-11545-7)
Supplement: Supplementary file 3 — Additional file 3: Supplementary Figure 1. Schematic representation of the organisation of the human health sector in India, showing the network of actors and information flow. Source: Planning Commission of India (2011) as cited by Gupta and Bhatia (undated). The Indian Health Care System. Available online at https://international.commonwealthfund.org/countries/india/ (Accessed on 19/02/2020). [file 12889_2021_11545_MOESM3_ESM.docx]

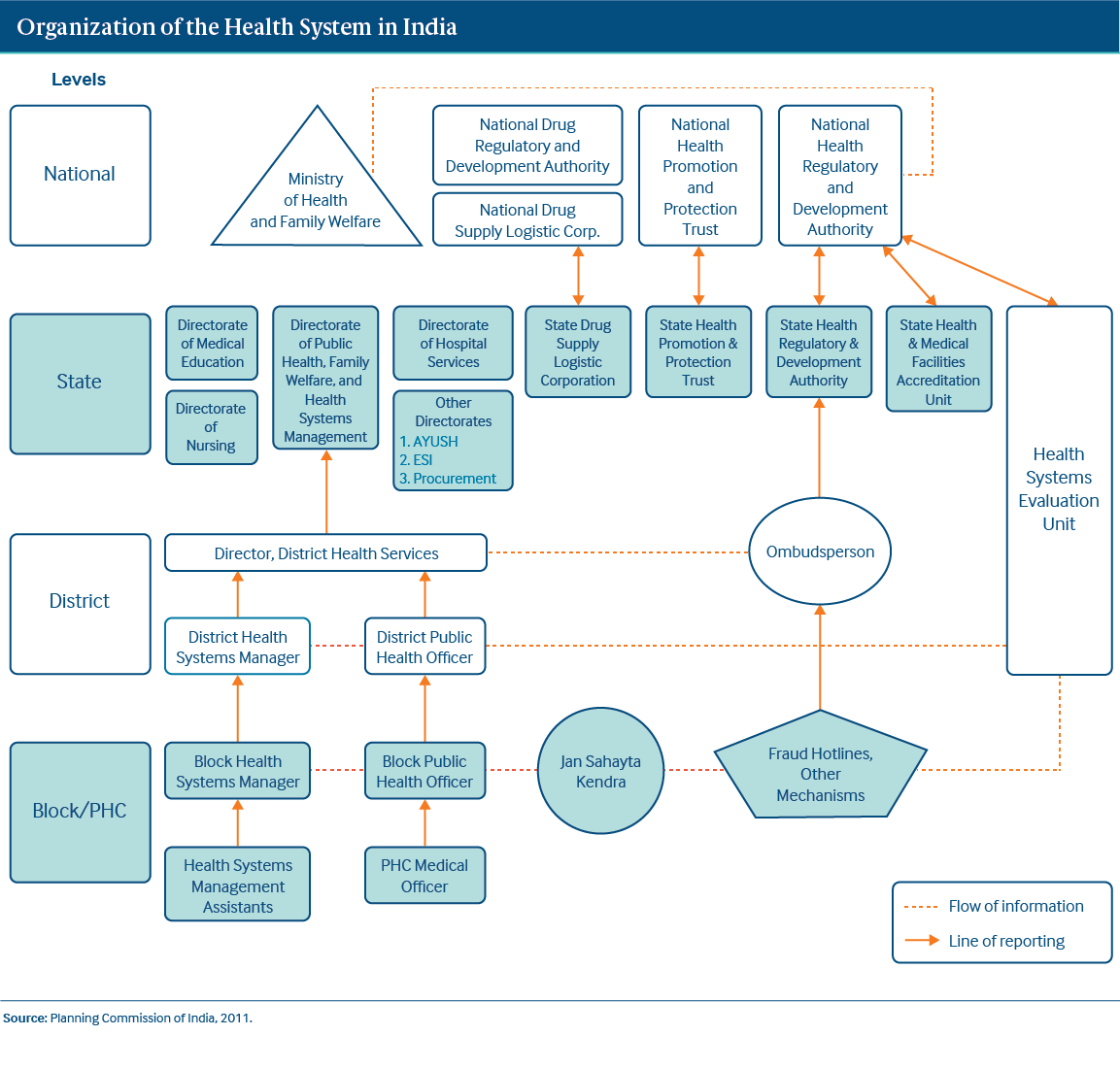


**Supplementary Figure 1.** *Schematic representation of the organisation of the human health sector in India, showing the network of actors and information flow*. Source: Planning Commission of India (2011) as cited by Gupta and Bhatia (undated). The Indian Health Care System. Available online at <https://international.commonwealthfund.org/countries/india/> (Accessed on 19/02/2020)
