# Supplementary material for: Operationalising the “One Health” approach in India: facilitators of and barriers to effective cross-sector convergence for zoonoses prevention and control
Source: BMC Public Health. 2021 Aug 6;21:1517. doi: 10.1186/s12889-021-11545-7 (PMC8342985; doi:10.1186/s12889-021-11545-7)
Supplement: Supplementary file 4 — Additional file 4: Supplementary Figure 2. Schematic representation of the organisation of the animal health sector in India, showing the network of actors and information flow. Source: Authors’ construct. [file 12889_2021_11545_MOESM4_ESM.docx]

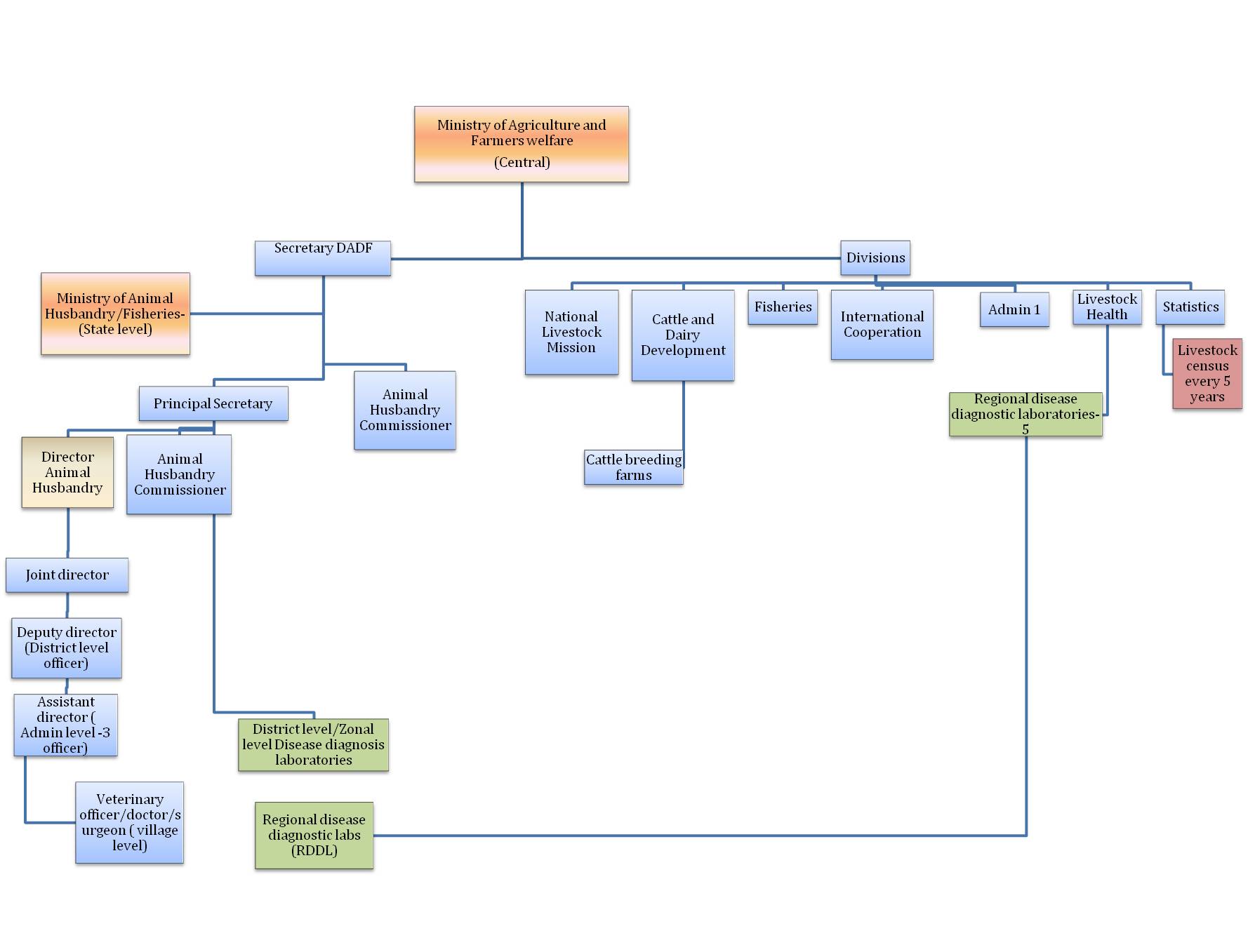


**Supplementary Figure 2.** *Schematic representation of the organisation of the animal health sector in India, showing the network of actors and information flow*. Source: Authors’ construct.
