# Supplementary material for: Operationalising the “One Health” approach in India: facilitators of and barriers to effective cross-sector convergence for zoonoses prevention and control
Source: BMC Public Health. 2021 Aug 6;21:1517. doi: 10.1186/s12889-021-11545-7 (PMC8342985; doi:10.1186/s12889-021-11545-7)
Supplement: Supplementary file 5 — Additional file 5: Supplementary Figure 3. Flowchart of the selection process of relevant documents. Authors’ construct. [file 12889_2021_11545_MOESM5_ESM.docx]

**Supplementary Figure 3.** *Flowchart of the selection process of relevant documents.* Authors’ construct
